# Medical feminism, working mothers, and the limits of home: finding a balance between self-care and other-care in cross-cultural debates about health and lifestyle, 1952–1956

**DOI:** 10.1057/palcomms.2016.42

**Published:** 2016-07-12

**Authors:** Frederick Cooper

**Affiliations:** 1 Department of History, University of Exeter, Exeter, Devon, UK

## Abstract

Post-war medical debates about the psychiatric consequences of married women’s economic behaviour witnessed far more divergence and collision between perspectives than has often been acknowledged. Practitioners who approached women primarily as facilitators of family health—as wives and mothers—were mistrustful of the competing demands presented by paid employment. They were faced by a growing spectrum of opinion, however, which represented women as atrophying in the confines of domestic life, and which positioned work as a therapeutic act. Advocates of work tapped into anxieties about family instability by emphasizing the dangers posed by frustrated housewives, shifting clinical faith away from full-time motherhood, but nevertheless allowing responsibilities towards husbands and children to continue to frame argument about women’s behaviour. Doctors, researchers and social critics, in this context, became preoccupied with questions of balance, mapping a path which sought to harmonize public and private fulfilment, identity and responsibility. This article traces this discursive shift through a series of conferences held by the Medical Women’s International Association during the early-to-mid 1950s, connecting debates in Britain with systems of broader intellectual exchange. It enriches and complicates historical knowledge of post-war relationships between medicine and feminism, at the same time as offering a conceptual and linguistic context for modern discussion about work-life balance and gender. This article is published as part of a collection entitled “On balance: lifestyle, mental health and wellbeing”.

## Introduction

Writing in the *New Statesman* on 14 February 2016, the feminist author and journalist Laurie Penny set out a persuasive array of reasons for women to think about the twice about the supposed benefits of heterosexual monogamous relationships. Echoing the pioneering sociologist Ann Oakley (1984: 91–96), whose research into domestic work and family relations during the 1970s and 1980s forged important pathways in feminist scholarship, Penny (2016) argued that the cultural and political structures intended to facilitate romantic love have often been more likely to stifle it. Her critique drew together a series of strands in women’s experiences of gender inequality, strands which are no less constraining for being less visible than the overt and unapologetic sexisms of the twentieth century:

Coupledom, for men, is not supposed to involve a surrendering of the self, as it is for women. Young men do not worry about how they will achieve a “work-life balance”, nor does the “life” aspect of that equation translate to “partnership and childcare”. When commentators speak of women’s “work-life balance”, they’re not talking about how much time a woman will have, at the end of the day, to work on her memoirs, or travel the world, or spend time with her friends. “Life”, for women, is envisioned as a long trajectory towards marriage. “Life”, for men, is meant to be bigger than that. (Penny, 2016)

Sixty years earlier, doctors, researchers and social critics were voicing comparable concerns about the effects of lifestyle on women’s selfhood and growth. Similarly engaged with questions of balance, commentators began to equate individual, familial and social health with a dual role for women, which divided their time and energy between home and workplace. As Claire Langhamer (2016: 13) has recently demonstrated in her article on the translation of women’s emotional labour from home to working environments, transformations in post-war debate at the intersection between emotion, health and occupational experience offer a rich resource for developing our understanding of present-day inequalities.

In Britain, in common with much of Western Europe and North America, a steep and steady rise in part-time employment for younger married women provoked and defied conservative anxieties about the stability and security of childhood and marriage (Riley, 1979: 99; Reynaud, 1983: 96; Lewis, 1988: 74). Writers and physicians who regarded motherhood as a full-time career rejected the possibility that women could reconcile the responsibilities and identities of mother, wife and worker without endangering the emotional development of successive generations (Spence, 1946: 23; Bowlby, 1951, 1953, 1958,). Their advice, feminist scholars have suggested, was guided by an unwavering blindness to the fatigue, frustration and isolation experienced by many housebound women (Ehrenreich and English, 1979: 203; Holdsworth, 1988: 122; Lewis, 1988: 22; Vicedo, 2011: 409; Alexander, 2012: 154). Contemporaries who took note of these worrying epidemiological indications tapped into longstanding connections between male health and productivity, welcoming and advocating paid work as a therapeutic or a prophylactic act (Miller, 1986: 143–176; Lutz, 1996: 259–282). Their enthusiasm, however, was tempered by concerns about the practical achievability of a vitalising equilibrium between public and private fulfilment. By the mid-1950s, the moral philosopher Mary Scrutton (1956) felt able to argue that working women had supplanted housewives as the healthier group, precisely because they contrasted the joy of nurture with individuality and self-discovery. While this marked a longer process in which workers and housewives exchanged places in cultural and clinical imaginings of psychiatric vulnerability, the margins between thriving and struggling as a working wife or mother were acknowledged to be paper-thin (Kanter, 1977: 61; Iglehart, 1980). Medical intervention and management, therefore, took on a new identity. Previously concerned with salvaging the health of predominantly working-class women whose double burden at home and work depleted their physical and psychological resources (Long, 2011: 147–152), some doctors positioned themselves as architects and gatekeepers of modern, aspirational lifestyles.

Female practitioners were at the forefront of post-war discussions about the medical implications of women’s working behaviour. This article follows the Medical Women’s International Association (MWIA), an umbrella organization for national associations and regional caucuses including the British Medical Women’s Federation (MWF), through a series of conferences and related publications in the early-to-mid 1950s. The meetings of the MWIA were one of many intellectual spaces in which women made use of sociological and medical interpretive frameworks to articulate dissatisfaction with domesticity, to dissent from suffocating models of motherhood, and to map a path which integrated a nascent clinical, ethical and practical case for work with widespread concerns about the breakdown of family life. Their deliberations repay careful study by challenging persistent simplifications of the role of post-war psychiatry in governing women’s attitudes to work, assumptions about the interplay between medicine and patriarchy that have been complicated by other research (Allen, 1986; Busfield, 1988). Social and cultural histories of Britain during the 1940s and 1950s have emphasized the nuanced and conflicting messages about work and domesticity which women encountered in their everyday lives, but have rarely recognized that medical narratives could be correspondingly complex (Holloway, 2005; Spencer, 2005; Beaumont, 2013, 2015). Clinical pluralism at the national level was fostered in part by exposure to arguments generated within other cultural contexts, as doctors and researchers made use of international networks, events and organizations to form and disseminate their ideas. The MWIA provided one forum for resistance to approaches which located psychosocial stability in the ossification of female identity. As divergent voices are brought in from the margins, their part in the alchemy of changing psychiatric and cultural knowledge is easier to recognize. It becomes possible, therefore, to identify the roots of contradiction in modern experience and discourse (Caproni, 2004; Clayton and Barton, 2011; Warren, 2015; Langhamer, 2016).

Following a substantive introduction to the formation, work, and self-conceptualization of the MWIA, the first section of this article investigates growing post-war apprehensions about the ill-effects of housework on women’s bodies and minds. Although industrial medicine provided a methodological and aetiological toolset to interpret housewives’ distress, the conceptualization of home environments as workplaces to be rationalized brought the limits of occupational analogies and the specificity of domestic experience into clearer focus. Meeting in Vichy in 1952, MWIA delegates used cyclical connections between physical ailments, psychological disorder, the rhythm and pace of household labour, and subjective feelings of unhappiness and frustration to question whether domesticity under any guise could provide the stimulation and self-fulfilment required by women (American Medical Women’s Association, 1953). The second section traces parallel concerns through debates about the experience and management of the menopause. Medical women had worked hard from the late 1920s to deflate deterministic representations of the “change of life” as an inevitable process of deterioration and crisis (Strange, 2012). Attendees at the MWIA 7th congress in Gardone in 1954 related endocrinological and psychological symptoms to sociological valuations of femininity and fertility, using the menopause as a barometer to gauge the emotional resilience afforded by conventional and non-conventional economic behaviour across the lifecycle (Medical Women’s International Association (MWIA), 1954). Finally, this article explores the emergence of a rhetoric of balance from the awkward collisions between conflicting emphases on self-care and other-care, connecting with Haggett’s (2016) introductory analysis of the linguistic and conceptual leitmotifs running throughout this collection. Members of the MWIA (1954), attempting to negotiate the implications of combining roles, drew attention to the interlinking legislative, individual, and cultural determinants of success and failure. Their accounts discerned danger in the advanced velocity of social change and a corresponding lack of formal supportive structures rather than the perceived erosion of “traditional” femininities.

## The MWIA

The engagements of MWIA members in debates about women’s working practices were, up to a point, self-referential. Surveys taken by the MWF in Britain reported that 57% of the affiliated doctors qualifying between 1933 and 1948 were married, with a lower rate among older women. Although women in medicine faced a number of professional and personal obstacles, the highest cause of occupational wastage was the difficulty involved in finding positions that could fit plausibly around family responsibilities (Medical Women’s Federation (MWF), 1958a). As in so many professions, this is a tension which has yet to be adequately resolved. Industrial action taken by doctors in 2016 against the imposition of new contracts by the Conservative Secretary of State for Health, Jeremy Hunt, has highlighted the disproportionate pressure the proposed measures place on female practitioners (Campbell, 2016). At a picket line in Bristol, one placard read “I strike because I want to be a doctor AND a mother one day” (Cooper, 2016). The MWIA offered an international forum for conversations about these collective struggles, as well as for the discussion of clinical challenges which were specific to female experience. Taking shape in the closing months of 1919 from a number of scattered groups and initiatives, the movement was envisioned as an overarching point of connection for national and regional groups of medical women (Ward, 2010: 1). Although only Britain and America had formed such federations at the time, one intention of inviting representatives from countries without existing networks was to stimulate their foundation.

Successive presidents and secretaries after 1945 elaborated a nuanced ethos of medical feminism which, in common with other post-war feminisms, played readily on constructed ideas of gendered difference to emphasize women’s exceptionality and justify their contribution to public works (Birmingham Feminist History Group, 2005). The association’s president, celebrated bacteriologist, and recipient of the Order of the Lion of the Netherlands for her activities in the Dutch Resistance, Anna Charlotte Ruys (1947) explained to a congress on post-war reconstruction that the complexities of healthcare in a changing world required medical women to become social workers too. This suggested not only an increased attentiveness to the life histories of their patients, but also positioned doctors as workers in and upon the social body, a role that experiences of nurture left them especially adapted to fulfil. “By the very fact that we are women”, she reasoned, it is “our duty to fit ourselves to take part in all the activities which regulate the life and future of our nation and mankind”. Ruys’ successor, Yolanda Tosoni-Dalai, expanded her approach in her first address to the organization in 1955. “Women doctors”, she wrote, “have the special task of studying and helping to solve the problems into which we have special insight through our sensitivity and femininity”. She expressed her hope that the council would endorse the official adoption of a hitherto informal motto, *matris animo curant*; “they cure in a motherly spirit” (Tosoni-Dalai, 1955: 4). Biographies of conference speakers frequently contrasted medical achievements with celebrations of fertility; in 1958, Tosoni-Dalai’s precis of achievement proudly announced that one of her daughters had also qualified to practice (MWF, 1958b). The MWIA, therefore, was an intellectual and emotional space where political, personal, and occupational ideologies, ideas and identities converged. With 2,300 British members in 1952, each in receipt of a quarterly journal, it disrupts depictions of post-war medical expertise as always for but not of femininity, and as necessarily running along anti-feminist lines (Ward, 2010: 74).

## Interpreting domestic distress

In her sociological autobiography, *Taking It Like A Woman*, Ann Oakley located her own experiences of domestic depression amongst a far wider “guilt, anger, loneliness, frustration, the dehumanization of women, our forfeited selves”. Betty Freidan, she explained, called it *The Feminine Mystique*:

And she called it that a long time before many of us knew there was anything wrong besides ourselves. Antidepressants, tranquilisers, obscurantist psychoanalysts and busy GPs: these represented techniques of adjustment that appeared reasonable because we thought individual adjustment was just exactly what was needed. (Oakley, 1984: 70)

The principle of adjustment, as Oakley describes, rested on the identification of an intrapersonal inability on the part of the patient to reconcile their conscious and unconscious needs with their perception of reality. Doctors such as F.P. Haldane (1950) used this framework to suggest that women who found domesticity constraining were too psychologically rigid, clinging to old expectations rather than allowing themselves to be comfortable in their present circumstances. The use of drugs to medicate housewives, denuding women’s discontent of its political connotations, has been a particular focus of investigations into the social consequences of therapeutic technologies; a powerful image of control because it represented the extension of medical anti-feminism directly into women’s bodies (Metzl, 2003: 17; Herzberg, 2009: 81). Doctors, within this characterization, played a double role in the construction and policing of post-war domesticities. They helped to construct them by investing women with inflated responsibility for the psychological needs of children and men, thus making a scientific fetish of motherhood and marriage (Mead, 1954: 477). Dissatisfaction with these condoned roles was then interpreted as a psychiatric problem, confirming a prevalent medical and cultural stereotype of female emotional fragility (Broverman *et al.*, 1970: 1–7; Hirshbein, 2010; Jackson, 2015: 125). Nuanced revisionism has disrupted some of these images, questioning the extent of felt distress amongst housewives and the impact and motivation of clinical attempts to govern women’s behaviour (Wilson, 1980: 189; Giles, 2004; Langhamer, 2005; Gill, 2007; Haggett, 2016; Halliwell, 2013: 150; Thomson, 2013: 104).

It has been less clear, however, that some doctors were prepared to reject compartmental approaches to individual illness, making connections between health and lifestyle that implied political rather than personal change. Inter-war studies of housewives’ illnesses used women’s suffering in specific economic and geographical settings to structure explicit critiques of urban poverty and suburban anomie, recommending action to improve standards of living and to provide cultural centres and stimulate community ties in new, alienating estates (Taylor, 1938; Spring Rice, 1939). Observers treading similar ground in the late 1940s, although remaining sensitized to environmental and personal pressures, were beginning to approach housewives as a distinct occupational population with shared medical experiences. Surveys taken by doctors in Britain offered a bleak clinical picture. Stella Instone, a physician at the New Sussex Hospital, and Dagmar Wilson, a nutritional expert at the Institute of Social Medicine at Oxford, each recounted widespread unhappiness, angst and fatigue. Of the 61 housewives Instone (1948: 900) studied, she found only 12 who had “no worries”, 45 with “some significant anxiety” and 4 who were “anxious about everything”. Wilson (1949: 140), from a sample of 194, found that 79% (153) reported “vague symptoms of tiredness, anxiety and depression”. Addressing the MWIA in Vichy in 1952, a paediatric specialist, Zaida Ericksson-Lihr (1953: 54) emphasized that researchers occupied with the problems of women in the home were responding to similar crises in their countries of origin. “Women doctors’ consulting rooms”, she explained, “are filled with distraught and confused housewives, seeking help”.

## Household fatigue and industrial medicine

Pressure to direct concerted attention towards the health of housewives had been building within the MWIA since 1948, when the problem had been raised during a meeting of their inner council. Regional symposia in Lillehammer in the same year and Aulanko in 1949 had culminated in a scientific session on the “pathology and hygiene of housework” at the yearly caucus in Philadelphia in 1950, with delegates appropriating methodologies from industrial medicine to describe and assess the potential hazards of domestic environments (Ward, 2010: 68). As Hepler (2000: 106–107) explains in her history of motherhood and occupational health in America, *Women in Labor*, the Philadelphia workshop understood housework as intrinsically important and necessary work for women, and set out to improve the physical conditions under which it was performed. Discussions in Vichy two years later incorporated elements of this approach, but also highlighted the inability of industrial analogies to fully illuminate the causes of ill-health.

Zaida Ericksson-Lihr described an archetypal middle-aged housewife who presented with symptoms of back pain, but tested negatively for nephritis, cystitis, gynaecological problems and slipped discs. An aetiological indication could be built by taking thorough descriptions of physical routines, and details such as the relative height of tables and kitchen surfaces. It was through painstaking reconstruction of their patients’ labour that doctors could isolate the source of their complaints. “It may be poor equipment in the home; it may be poor arrangement of the household facilities; it may simply be too many backbreaking hours of intensive, hurried work for the family for too many years” (Ericksson-Lihr, 1953: 54). Her examples highlighted tensions which delegates at Philadelphia had been unable to convincingly reconcile (Hepler, 2000: 106–107). As Rhodri Hayward (2007: 51) recognized in his study of inter-war representations of household alienation, assumptions made by doctors about the uptake and use of labour-saving devices went frequently wide of the mark. Positing better equipment as a solution to housewives’ problems, too, tied health to consumer culture in ways which ran counter to earlier critiques of suburban materialism. Although sociologists in the mid-to-late 1950s such as Alva Myrdal, Viola Klein and Judith Hubback traced a broad improvement in the nature of domestic work across America and Western Europe, the generation of women writing in Britain in the 1940s emphasized that there remained many tasks which job design and household technologies were unable to reduce or simplify (Luetkens, 1946: 39; Brown, 1948: 10–11; Myrdal and Klein, 1956: 38; Hubback, 1957: 60). Elite observers noted the never ending nature of housework and the piecemeal approach that many women adopted, drawing the uneasy conclusion that the problem lay in women’s own attitudes as much as the jarring composition of work.

It was precisely the use of occupational health frameworks to explore the uncharted terrain of the home which highlighted the limitations of industrial vocabularies. Ericksson-Lihr’s co-panellist in Vichy, Doris Odlum, articulated one aspect of the problem in her report to the conference. A pivotal figure in the British Medical Association and the European League for Mental Hygiene, Odlum (1953: 62) confirmed that scientific enquiry into housework was “long overdue”. It was well known, she told her audience, “that in practically all countries women are still working under most unsuitable conditions, which in many cases are having unfavourable effects on their health from both the psychological and the physical point of view”. Reviewing British research, Odlum cited published studies and ongoing research by Dagmar Wilson, by the Women’s Group on Public Welfare, and by the sociologist Judith Hubback. According to Hubback (1957: 60), the biggest obstacle for reformists was the contradiction between homes as necessarily personal domains and as sites for measurement and rationalization. Housewives, Odlum complained, had “rigidly clung to outworn and unsatisfactory methods and conditions even when they were given the opportunity to improve them”. Simultaneously, they had rarely taken steps to organize to ameliorate their own circumstances (1953: 61).

Although demonstrating faith in the potential advantages of industrial techniques, Ericksson-Lihr (1953: 55) recognized an interrelated aspect of women’s discontent which drew sharper distinctions between housework and outside employment. “How about the psychic troubles of the housewife”, she wondered. “Are they real or only imaginary? Did the long working hours by day and night, the hectic hurry, the economic difficulties, the loneliness and lack of appreciation, upset her balance?” Her approach, taking a parallel psychological inventory to detect signs of social isolation, restlessness, poor sleep, “monotony of life” or symptoms of an “inferiority complex”, hinted at existential difficulties which evaded resolution even by successful attempts at job design. The seclusion in which many women worked, in part a result of rising geographical mobility, but also attributable to spreading middle-class associations between privacy and respectability, carried psychiatric connotations that were just beginning to be seriously explored (Halmos, 1953). In addressing appreciation for household labour and pathological feelings of inferiority, Ericksson-Lihr was connecting unhappiness and fatigue with subjective perceptions of status. Claire Langhamer’s observation that post-war celebrations of domesticity often masked a steep decline in the prestige of domestic work was reflected in concerns voiced about women’s health across the 1940s (Riesman, 1950: 300; Langhamer, 2005: 359). One left-wing social researcher and feminist activist, Amber Bianco White (1941: 93), implicated the devaluation of housework in heightened experiences of anxiety. As long as housewives thought of what they did as an “unworthy, inferior, miserable sort of occupation”, it was impossible for them to derive any psychological rewards from their exertions. The principal difference between outside work and the “domestic salvage” that housewives performed, according to another critic, was that the latter had “surrendered its inherent dignity”. The toll that this took on women was the “price of home” (Luetkens, 1946: 111, 109). This was a world, fundamentally, that Ericksson-Lihr (1953: 54) surmised had “grown too narrow.” Housework alone, she concluded, “is not enough to make most women happy”.

Improving the conditions and status of domestic labour, therefore, could only take women so far. For her part, Ericksson-Lihr was giving voice to a rising international acknowledgement that, in the words of Ena Brown (1948: 5), the “expectation that every type of woman ought to find within the home satisfaction for all her needs, mental, physical, and emotional is an assertion so sweeping as to show little understanding of the conflicts which may be involved”. The inclusion of “most” women in this category shifted the parameters of debate. Like Odlum, Ericksson-Lihr was making use of a language of universal psychological requirement that moved beyond social and national contexts and invalidated medical responses aimed at individual adjustment (Lewis, 1953: 116). Their colleague, a leading French writer on youth, sex and motherhood, Germaine Montreuil-Straus, drew these strands together during a corresponding paper on the “psychosomatic aspects of housework”. Married women’s over-strain, she explained, was grounded in an “emotional, psychic, and mental disequilibrium”. Low-status labour which lacked temporal and spatial definition intensified any fatigue imposed by its physical performance (Montreuil-Straus, 1953: 60). In common with critics of feminism, Montreuil-Straus argued that women’s dissatisfaction in the home had to be understood as the product of a historically contingent tension between raised educational and socioeconomic expectations and lowered valuations of traditional behaviour (Lundberg and Farnham, 1947). “Women who are growing more and more conscious of their own personalities and aptitudes and possibilities”, she argued, “feel very strongly that their standard of living has been drastically lowered and their inability to make the necessary adjustments results in a more or less permanent loss of physiologic and nervous stability” Montreuil-Straus, 1953: 60–61). Rather than implying the need for a retrenchment of conservative values, the connections she made between women’s illness and cultural transformation were fundamentally positive. Felt distress, itself a by-product of social and political progress only half-realized, could provide the impetus to push forward to a fairer world.

In taking their interpretations of household pathology beyond simplistic connections between the physical workplace, tiredness and pain, the speakers at Vichy made use of an aetiological model of circular distress that relied upon the conviction that unhappiness, perception and subjectivity were as much—if not more—to blame than workloads which were objectively debilitating. It became plausible to conceive of outside work as a solution rather than an added problem because the stimulation and fulfilment it provided outweighed the additional effort involved, dissipating the feelings of frustration and worthlessness that lay behind fatigue (Zweig, 1952: 24; Hubback, 1957: 60; Jephcott *et al.*, 1962: 108; Wilson, 1980: 205). Although they presented imposing critiques of full-time domesticity from a medical perspective, Ericksson-Lihr and Montreuil-Straus also demonstrated the continued pervasiveness of assumptions about the role of women in maintaining the integrity of family life. Pushing back against clinical and moral arguments which urged that healthy marriage and motherhood required women to stay in the home, Montreuil-Straus (1953: 61) suggested that psychosomatic over-strain in young, unsatisfied housewives could itself lead to that most ominous of phenomena, “family instability”. The implication of her argument, that family health demanded the diversification of women’s interests, left the assumption intact that female behaviour would continue to be judged in instrumental terms. Ericksson-Lihr concurred. Although she described a “revolutionary project” in which the “life role of the woman” was guided beyond cooking and mending towards immersion in creative work, the ultimate aim was nevertheless to facilitate the personal growth necessary to “bring up physically and mentally healthy children in a stable and happy family environment”. Earlier elements of her presentation had touched upon just how precarious this balancing act could be. Without assistance in childcare and housework, she cautioned, “sooner or later even the most capable woman is lost” (Ericksson-Lihr, 1953: 58).

## Surviving the menopause: psychological resilience and personality formation

Reassessing the relationship between working motherhood and child and adolescent health in 1963, Simon Yudkin and Anthea Holme (1963: 180) recognized that debate about the illnesses of housewives had centred around two particular stereotypes. The first of these were women who, through prior experience of education or work, found household duties limiting and frustrating. For the most part, this was the category addressed by attendees at the MWIA conference in Vichy. The second type were those who may have been able to find fulfilment in motherhood but were unable to cope with the adolescent independence of their children, a crisis in purpose which dovetailed into a decline and loss of reproductive function and negative valuations of ageing female bodies. The influential postwar feminists Myrdal and Klein (1956: 39) envisaged this as a “phase of acute emotional crisis” characterized by feelings of emptiness, serious discontent, and the heightened possibility of nervous breakdown. Their solution, a tripartite sequence in which education was followed by motherhood and then paid work, echoed arguments in favour of the wartime mobilization of menopausal housewives by emphasizing the psychological worth of new responsibilities (Medical Women’s Federation, 1943: 512; Lewis, 1990: 170). As Julie-Marie Strange (2012: 697) has identified, doctors in the MWF were still struggling in the 1940s to gain recognition for evidence from inter-war surveys, which challenged depictions of the menopause as inevitably disabling.

When the MWIA turned their attention to the menopause in their conference on the shores of Lake Garda two years later, it was clear that the complexities of women’s lifestyles were still weighing heavily on the minds of some of the delegates. Judith Houck (2006), in her nuanced study, *Hot and Bothered*, has explored in detail the effects of feminist and medical interpretations of the menopause in shaping and contesting wider assumptions about women in modern America. Although she acknowledged that mixed messages about work and domesticity were transmitted to women from a number of sources during the 1940s and 1950s, her work constructed 1963 as a watershed between reaction and dissent, in part because of the publication of Betty Freidan’s *The Feminine Mystique*. The important questions Houck (2006: 209) asks of sources in this later period, allowing her to make a sensitive appraisal of the ways in which second wave feminists connected sexism with symptoms and medical technology with liberation, resonate too with research presented by delegates in Gardone. Post-war writers who portrayed the menopause as opening up a new chapter for women by freeing them from their biological and racial function, she rightly observes, left gender roles during women’s fertile years implicitly uncontested (Houck, 2006: 90). While advocacy of work during the menopause, as one doctor argued, could be used to draw attention to the wider therapeutic possibilities it presented, it was also positioned as fundamentally consolatory (Van Andel-Ripke, 1954: 96). By using women’s experiences of the menopause as a yardstick for learned emotional health and resilience, however, members of the MWIA reflected a critical light backwards into women’s younger lives.

Building on concerns about domestic attrition voiced in Vichy, speakers in Gardone questioned the assumption that menopausal housewives could simply take on unfamiliar roles when they were no longer able to be active mothers. A neurologist and child developmental expert, Olga Van Andel-Ripke, presented a paper entitled “Mother and housewife in the climacteric”. Juxtaposing the physical changes that women underwent with the psychological challenges they faced in mid-life, she explained the consequences of tying self-worth to reproductive ability:

She is afraid of the coming years and dreads her own decline and inadequacy. Everything seems drab and gloomy, and even the realisation that she makes her family share her misery drives her more deeply into the narrow circle of self-pity and self-abasement in which she turns round and round without finding relief … In this atmosphere of false notions, mental distress, and feeling ill, the woman gets into a *circulus vitiosus* which involves the whole psychosomatic field. (Van Andel-Ripke, 1954: 96)

Van Andel-Ripke (1954: 93) contrasted housewives’ suffering with the experiences of employed women who, “sometimes after a brief period of imbalance”, usually found “a healthy stimulant to recovery in the love for or necessity of their work”. The dichotomy she constructed transcended debates about the healthiest use of time for women undergoing the menopause, exposing deep contradictions in the organization of women’s lives around femininity and fertility. The employed women she referred to were not those who had taken a job in middle age, but who had built up an inner resourcefulness and strength through a lifetime of work. Housewives, in contrast, were not just debilitated by domesticity on a day-to-day basis but could be permanently damaged and diminished by it. The problem, as some British sociologists argued, was one of atrophy (Williams, 1945: 97; Brown, 1948: 14; Hubback, 1957: 1–2). Van Andel-Ripke (1954: 96–97) described dejected women who, because of the “standstill in the development of [their] personality in and through marriage”, found their self-assurance and capacity for outside employment had been worn away, and were therefore unable to adjust to fulfilling work when they needed it most. As one commentator noted in *The Lancet* (1960: 1129), writers who presented the menopause as an opportunity for personal renaissance underestimated the danger of this “insuperable psychological block”.

In fact, Van Andel-Ripke (1954: 94) emphasized, “the menopausal complaints of those who do not feel at home in their work are often remarkably intense”. Her arguments represented a subversion of usual narratives about the menopause, endocrinology and the pathological female body (Hirshbein, 2010). The severity of symptoms was reconfigured as a litmus test for the emotional stability or lability of the woman in question, an equation connected explicitly with lifestyle. The conclusion that serious ill-health during the menopause was a consequence of the inability of traditional femininities to provide women with a coherent psychological foundation across the lifecycle formed a powerful call to change. It also suggested that opportunities outside of the home offered an alternative way of thinking about bodily change in relation to social expectations. The menopause was a traumatic process because women had been persuaded to value the characteristics it seemed to undermine, and to reject work as a source of protection and resilience. If the basis of femininity was negotiable rather than fixed, and was tied intimately to the performance of social function, then many of the problems associated with the menopause could theoretically be eliminated.

A shift in attention beyond inevitable biological processes and towards psychosocial understandings of the menopause in relation to constructed gender identity enabled a reappraisal of the specific challenges faced by working women. Inger Haldorssen, a Norwegian physician, delivered a brief survey of attitudes to the impact of the menopause on working ability. Despite “climacteric complaints” only accounting for a small proportion of sickness absence, she argued, doctors, employers and patients were still likely to regard the “dangerous years” as the beginning of a decline in professionalism, health and aptitude (Haldorssen, 1954: 99). According to a corresponding paper by a Canadian obstetrician, gynaecologist, and popular essayist on women’s health, Marion Hilliard, this meant that the greatest hazard posed by the menopause was social in nature. Hilliard (1954: 104) described three “common complications”; sudden haemorrhage, blood loss through excessive menstruation, and fatigue after amenorrhea. Working women, she argued, required particular help in overcoming these difficulties: “they are not serious. They are physiological dislocations but they may cost her job and ruin her future”. Although housewives were likely to undergo more distressing episodes which could even lead to institutional care, workers were placed in a precarious position by well-worn associations between hormonal imbalance and irrationality, and by stigma surrounding the uncontainable female body. The consequences of demotion or redundancy at this stage were catastrophic, and lay behind the development of far more serious problems in their aftermath. “We are born equipped”, Hilliard told her audience, “with a certain type of nervous system and emotional balance and must learn to live with it. At the menopause we find that we cannot control the depth of our reactions”. Careful medical supervision, therefore, was required to manage fluctuations in mood and to mitigate the galling effects of hot flushes and irregular bleeding. In her own practice, Hilliard prescribed small doses of sedatives such as amytal and phenobarbital, given during the day alongside relaxants such as transentin. These were intended to complement psychotherapeutic techniques, as well as adjustments to oestrogen levels. Nothing could be tolerated, she argued, which undermined the perception of efficiency or self-confidence (Hilliard, 1954: 106).

The close medical management of menopausal symptoms was reconfigured as a feminist technique, therefore, to safeguard women’s careers during a critical psychological and sociological phase. Widely expected to take up presidency of the MWIA but for her early retirement in 1956 and untimely death in 1958, Hilliard presents a complex figure for historians to interpret. Houck (2006: 117; pp. 122–123) presented her as a reactionary figure who depicted menopausal women as manipulative and self-indulgent, compared them to adolescents, and encouraged them to maintain physical relationships with their husbands even in the event of severely diminished sexual urges. Conversely, her collection of essays in *Chatelaine*, a Canadian women’s magazine, have been construed as prescient and provocative warnings about the fragility of women’s health in traditional roles (Mendes, 2010). Published in Britain as *A Woman Doctor Looks at Love and Life*, Hilliard (1958: 109) asked a number of searching questions of menopausal wives and mothers. “Does she know what life is about, I think to myself. Does she have a core of serenity, derived from the knowledge that she is a capable, coherent human being?” According to her analysis, this was exactly what housewives were missing. Without it, their sense of uselessness could spiral downwards into alcoholism, drug use, or mental illness. With it, and with “some consuming occupation, whether it is a study of fourteenth-century Chinese art or an office to manage, she isn’t in much danger of being shattered by what is happening to her physiology” (Hilliard, 1958: 112). The loss of fertility, not as a biological fact but as the basis for social identity, was the “deep dark water under the thin ice of a married woman’s composure” (Hilliard, 1958: 103).

In common with Zaida Ericksson-Lihr and Germaine Montreuil-Straus, Hilliard cautioned that domestic unhappiness had a suffocating effect on family life. She painted a vivid portrait of the women who, remembering “the conviviality of the office they left for motherhood”, took out their frustration on their children. “She prides herself on being a good mother because she isn’t working: in her heart she must know she is a terrible mother”. Although women who failed to balance their work with their family responsibilities were also “suffering a defeat on all fronts”, it was recommended that they try (Hilliard, 1958: 110). “Women need to work to gain confidence”, she wrote. “Women need to work in order to know achievement. Women need to work to escape loneliness. Women need to work to avoid feeling like demihumans, half woman and half sloth” (Hilliard, 1958: 104). Just as sociological insight helped to frame clinical understandings of domestic labour, medical recommendation was becoming embedded in the lexicon of British sociology. Researchers unearthed instances of doctors prescribing work to housewives, usually as an antidote to nervous illness (Zweig, 1952: 24; Cartwright and Jefferys, 1958: 159; Klein, 1959: 35; Jephcott *et al.*, 1962: 109). Viola Klein (1960: 39) and Yudkin and Holme (1963: 46–47) each reflected that the frequency of women who reported looking for paid employment as a result of medical direction implied a growing recognition of its “therapeutic value” among practitioners.

## Health in the balance

Explaining the advantages of outside work for frustrated and fatigued housewives in the mid-late 1950s, Judith Hubback warned that the type of job women were able to consider was “limited to those which will not ask for an excessive amount of her time, loyalty, and nervous energy”. She encouraged her readers to think of womanhood, characterized by socio-cultural obligations rather than inherent frailty, as a disability. “It is essential”, she argued, for female workers to “come to terms with this disability, as she would have to, for example, with deafness or blindness”. The key to happiness and health was to “do the right amount of outside work, the amount which will restore and not impoverish them. It is a question of finding the balance” (Hubback, 1957: 93, 149). Intrapersonal balance between different sources of fulfilment and identity was intertwined, as Myrdal and Klein (1956: 28–29) argued, with the need for “a more stable equilibrium between the demands of the community and the needs of the individual”. Langhamer (2016: 3) has described the appeal to emotion which accompanied attempts to convince married women to move back into industry in the aftermath of the Second World War: “The health of children, husbands, nation and (more rarely) women themselves, was held to rest on the correct deployment of female labour outside the home. Too little and the economy would falter; too much and society would suffer”. While economic pressures and arguments certainly structured debate, medical discourses around familial and social breakdown played a significant part on both sides of the argument. The deputy director of the Tavistock Clinic, Dicks (1955: 297), warned that women were being caught “between care and independence”. Doctors in the MWIA were among those who came to the conclusion that these were not just compatible, but essential to one another.

In the September of 1956, members of the association met at an extraordinary general assembly in Burgenstock, Switzerland, to discuss the role of medicine in managing the difficulties faced by married workers and the potential implications these had for the health of their families. Convocations of the World Federation for Mental Health and the European League for Mental Hygiene (ELMH) had each addressed identical themes in 1955, as attention to the issue within international medical networks gained ground. Odlum, the British representative who had spoken in Vichy on the difficulties of applying occupational methods to domestic work, had attended both. Reporting on the deliberations of the ELMH, she drew together the commonalities in approach between that organization and the MWIA audience in Burgenstock. ELMH delegates had made similar observations about the medical impact of domestic boredom, linking health with occupational emancipation (Odlum, 1956: 26). Child delinquency, they argued, was far more likely to occur in “problem families” whose parents were “too inefficient” to go out to work, a supposition which illustrated the continued traction of inter-war connections between poverty, heredity, morality and cleanliness (Riley, 1979: 98; Welshman, 1996; Starkey, 2000). Odlum concurred with Paul Sivadon, a prominent French psychiatrist and mental hygienist who had delivered the keynote lecture to the ELMH. For Sivadon, outside work “usually made it possible” for a woman to “carry out both her maternal responsibilities and her role as a married woman more satisfactorily” (European League for Mental Hygiene, 1955: 785; Odlum, 1956: 21). The most important factor, he argued, was the quality of the personal relationships women were able to sustain with their children and husbands. If these were good, then they could weather periods of absence. If they were bad, then the situation was unlikely to be improved by continuous exposure to one another, particularly if conscious or unconscious resentment towards domesticity played a part (European League for Mental Hygiene, 1955: 785). Frustration could manifest itself, Odlum reasoned, in increased irritability with children and recourse to systematic and arbitrary punishment (Zweig, 1952: 75; Odlum, 1956: 22). While some psychoanalysts, guided by the work of John Bowlby, claimed that “deprivation—of parental love—and even deprivation of the love of bad or incompetent parents—makes a super-hash, a kind of witches’ brew”, the assertion that children could be deprived of love by ever-present parents formed an effective counter to their arguments (Editorial, 1951: 1165; Van der Horst, 2011; Thomson, 2013).

Odlum (1953: 446) had previously expressed caution that the psychological requirements of young mothers were being obviated by those of their children, reminding readers of *Family Doctor*, the magazine of the British Medical Association, that “the wishes, and even to some extent the needs, of the baby must be modified to fit in with her needs too”. Children in receipt of constant maternal attention were by no means guaranteed healthy psychological development, but could fall prey to a divergent strain of pathological motherhood, the overbearing “mom” who recurred frequently in American debate (Rioch, 1955: 53; Odlum, 1956: 25; Lunbeck, 2012: 55). A focus on children who experienced a deficit of mother-love obscured the problems of those who were enveloped by it; “smother-love” could be just as dangerous to the unformed psyche (World Federation for Mental Health, 1955: 54). Odlum’s scepticism of Bowlbyite determinism found further confirmation in evidence presented to the MWIA by a French child psychiatrist and expert on adolescent delinquency, Suzanne Serin. Drawing on decades of experience giving advice to juvenile courts, Serin (1956: 32–33) described psychologists who conflated good motherhood with “la femme au foyer” (the housewife) as “zélateurs” (zealots). The connections they made between working mothers and serious emotional disturbances had no reflection in the medical and legal cases in which she had been involved. Anti-work critics, she maintained, had been systematically unable to demonstrate that the work of the mother beyond the home had significant adverse effects “sur sa santé, sur son équilbre, sur la santé ou l’équilibre du mari, de ses enfants ou en general sur la stabilité du couple” (on health, on balance, on the health or the balance of the husband, her children or in general on the stability of the couple). In emphasizing the reduced intellectual traction of maternal deprivation theory outside of Britain, contact with critics such as Serin disrupted the supposed universalism of knowledge about childhood development. By shifting the arguments of writers such as John Bowlby away from the value-neutral spaces they claimed to inhabit, it became possible to detect, as Mead (1954: 477) observed, a “new and subtle form of antifeminism”.

Men, Odlum (1956: 28) thought, were taking the revolution in women’s employment in surprisingly good humour. Concerns about the medical consequences of attendant shifts in marital power dynamics, although causing some writers (Hutchin, 1960; Cohen, 1960; Morris, 1961) to rediscover the salutogenic properties of masculine dominance, were losing ground to a broad acceptance of the holistic psychological benefits of a softened system of patriarchal authority (Mace, 1948; Bliss, 1953; Casson, 1959; Chesser, 1959; McCarthy, 2016). It was the inevitability of damage to family life that Odlum and Serin refuted in their presentations. Although Odlum argued that working women could enjoy improved relationships with their husbands and children, both acknowledged that their experiences lay on a long and complicated spectrum, mediated by a variety of structural, environmental and individual factors. Women could still harm their families by becoming overwhelmed by conflicting responsibilities or identities, leaving them unable to carry out their function as wives and mothers. A German sociologist writing for a series entitled “The New Democracy”, Charlotte Luetkens (1946: 125) posed the following question: “Now that a woman is faced with an almost unlimited variety of choices, since her relationships, activities, and duties have multiplied, why should we expect a woman’s life to be free of conflicts, tensions, and unsatisfied desires?” Although these pressures themselves presented serious psychiatric challenges, there was a shared sense among the MWIA that they were surmountable by social and medical measures (Serin, 1956: 34; Ruys, 1956: 39). Conference proceedings appearing in the *British Medical Journal* highlighted the attitudes of the “Far Eastern” working group. Delegates from India and China, while admitting little experience of the problems under discussion, claimed to be “looking forward to the day when [they] would have” (MWIA, 1956 b: 1297). This comparison constructed complexity in women’s lives—even potentially pathological complexity—as a component of western social progress, set apart from the experiences of countries supposedly slower to modernize.

Only one of the speakers struck a discordant note. A paediatrician who founded the Institute for Mental Health in Childhood in Zurich, Marie Meierhofer (1956: 12–13), concluded that married workers placed themselves at undue risk of “un surmenage” (overwork/fatigue). Less than half of married workers in Zurich, she argued, experienced good health. Instead, they suffered from headaches, circulatory disorders and back pain, accompanied by guilt, tension, loss of coping, and pervasive feelings of inferiority. This self-perpetuating range of disorders destabilized “l’atmosphére familiale” (the family atmosphere). When her arguments were challenged in discussion by the remaining speakers, a number of delegates, and a few of her Swiss colleagues, Meierhofer explained that the ethical and medical dimensions of the question were negated by the national political context. Swiss legislative practice was presently organized around encouraging women to stay at home, raising men’s wages at the same time as providing financial incentives in the form of grants and tax relief to full-time mothers (Ruys, 1956: 37). Practical measures to assist women who went to work, therefore, received little support. In this inhospitable atmosphere, she argued, the possible benefits of work to women were largely unable to be realised; they were abstract, they held no substance. Other members, disappointed by her unwillingness to pay lip service to the worth of work in the face of what she regarded as discouragingly prohibitive circumstances, characterized her position as a “negative” stance which looked on the “darker side” (MWIA, 1956a, b: 1297).

In contrast to Switzerland, Scandinavian legislative measures were broadly supportive of women’s choices. Myrdal’s (1941) study, *Nation and Family*, had recast the question of married women’s right to a job as one of working women’s right to a family. An advocate of contraception and women’s health specialist in Copenhagen, Inge Jespersen, reported to the MWIA on innovations in Denmark, Norway and Sweden. Women were empowered to balance work and family without negative implications for either health or family life, she explained, by a series of initiatives; prohibition of night work; maternity leave; flexible working hours; job security on pregnancy or marriage; nurseries and crèches; permissive taxation; and means tested domestic help for working mothers. The problems women faced, however, could not be said to have been entirely solved, necessitating further legislation, increased male participation in housework and parenthood, and changes to sexist employment cultures which disadvantaged all workers, married and single (Jespersen, 1956: 3–4). Although post-war British policymakers certainly failed to adequately anticipate women’s changing needs (Riley, 1987), Odlum (1956: 20–22) described a cultural and legal situation which, while falling short of Scandinavian state feminism, yet allowed for more optimism than the Swiss report. Summing up proceedings, Ruys (1956: 36) emphasized that it was “not so much that the woman works outside her home, which causes mental or physical breakdown of the wife, but the special circumstances of the case”. These were not social and legislative wallpaper, a background to the personal and psychological balancing acts that working wives and mothers had to perform. They were the ingrained structural mechanisms which, in conjunction with important factors such as individual disposition, family health, living conditions and marital relations, governed whether women thrived or floundered. In the arguments of each of the delegates—including, to a degree, Marie Meierhofer—the negative consequences of work were reconfigured as culturally and politically conditional, and, therefore, as potentially responsive to medical and social management.

Stepping outside of the nuanced ethical and medical case for work presented by the majority of speakers, both Odlum and Ruys emphasized that doctors in and beyond the association would be increasingly required to assist women in navigating the complexities of dual roles whether they approved of the development or not. For Odlum (1956: 27), the propulsion of women into work as a means of escape from the isolation and dissatisfaction of full-time domesticity had become irreversible and irresistible. Ideological resistance rooted in prejudice, therefore, could only ever obfuscate rational debate. Outdated objections needed to be put aside in order for women and experts to “devise the most satisfactory methods of adapting family life to the new situation”. Echoing recurring themes identified by Jackson (2013: 62) in discourses on nervous illness and stress in the late nineteenth and early twentieth centuries, the problem was located in an imbalance between the pace of transition and the ability of individuals and communities to quickly adapt:

Like all rapid social change it gives birth to conflicts and could therefore be pathological. In the interests of the mental health of the family it is essential to find solutions to establish an equilibrium between the traditional demands and these changing tendencies in order to preserve the stability of the family group and the satisfactory development of the personality of the child. (Odlum, 1956: 25)

Ruys developed Odlum’s analogy, situating the medical consequences of emancipation as “part of the dynamic process of the evolution of mankind”. Female vitality, she argued, was growing with an “astonishing rapidity” which “cannot be arrested”. Like a river bursting from its banks, it could cause damage, but was “also fertilizing new grounds” (Ruys, 1956: 36). The purpose of the conference in Burgenstock was to put this damage into perspective, counteracting the deep professional and emotional impressions made on doctors who had witnessed individual women “break down under a double task of family duties and work”. The juxtaposition of female experience across national contexts threw commonalities and differences into sharp relief. Comparison invited practitioners to pay closer attention to the specific factors framing women’s behaviour in their own countries, but also encouraged a sense of shared struggle, of health and illness as problems of gender. Identities as doctors, feminists and women again converged, as Ruys (1956: 39) closed the conference. Members of the MWIA, she emphasized, “only have the right to welcome the shift we are witnessing if we have done our utmost to relieve the strain of those on whom the burden is too heavy”.

## Conclusion

The work of the MWIA to contest and criticize reductive and inflexible models of femininity formed one strand in hitherto overlooked feminist medical discourses in the 1950s. Although patterns of resistance to the clinical rationales underpinning a rigid sexual division of labour have been identified, they have usually been located within the social sciences or in women’s own actions (Ford, 1953: 394). The result has been that medical approaches have seemed monolithic in their conservatism, when they were characterized by dissent, debate, and diversity. MWIA members, in their discussions of the lived experience of household labour, the need for strong psychological foundations in the mid-life, and the complexities of personal and practical balance, were rehearsing concerns about gendered predisposition to illness which gathered momentum in Britain as the decade turned (Carstairs, 1963). Although Haggett (2016: 103) has demonstrated that both women and doctors were far more likely to implicate disordered familial relationships in the development of neurosis, contemporaries also emphasized the pressure that domestic isolation placed on intimate emotional bonds (Brown, 1948: 8; Dicks, 1955: 296; Mogey, 1956: 156; Myrdal and Klein, 1956: 148–149; Odlum, 1956: 22).

Prefiguring arguments made by second wave feminists, women in the home were depicted as incomplete or “damaged selves” for whom paid work was essential for resilience or recovery (Johnson and Lloyd, 2004: 27). The blurred intersection between femininity and disorder in psychiatric discourse, by this mechanism, was in the process of reconstitution from a biological to a sociological basis. Female weakness and lability, in this imagining, were products of enervating domestic lifestyles, and the interwoven systems of emotional identity and sexual hierarchy which they promoted. The conflation of productivity with personal growth, in practice, left few with the time or energy to pursue the “study of fourteenth-century Chinese art” (Hilliard, 1958: 112). Leftwing critics in particular have destabilized the assumption that moving away from the home necessarily represented either improvement or choice for many women. Although a psychosomatic interpretation of domestic fatigue allowed female distress to become politicized, it also elided the experiences of working-class women who often faced unreconstructed household labour, husbands whose alienation at work translated into patriarchy at home, and jobs which afforded little in respect or individuality (Rowbotham, 1973; Pollert, 1981; Bradley, 1989; Stanley, 1989; Buswell, 1992; Warren, 2015).

Steeped in linguistic conventions which used balance as a signifier for health in emotional (Ericksson-Lihr, 1953: 55; Montreuil-Straus, 1953: 60), endocrinological (Van Andel-Ripke, 1954: 93; Hilliard, 1954: 106) and familial contexts (Serin, 1956: 33), the concept of “equilibrium” between “traditional demands” and “changing tendencies” seemed uncomplicated. It reproduced unconscious sexisms, however, in moving women towards an ideal model of implicitly neutral masculine lifestyle, which was itself predicated on their continued emotional labour (Casson, 1959: 159; Morris, 1961; Langhamer, 2016). The “traditional demands” which worked as a counterweight to newly visible working identities signified an uncomfortable continuity in the valuation of women as supporting actors in other people’s lives. While MWIA delegates subverted reactionary anxieties about working women and family pathology, they nevertheless allowed responsibility for the health of husbands and children to continue to frame debate about women’s behaviour. The functionalist vision of home as a haven for male workers was left unshaken by the post-war feminist construction of work-places as similar sanctuaries for housewives, writing women’s imbalance directly into men’s success and fulfilment. When Penny (2016) explains that the “life” in “work-life balance” translates for women today as “partnership and childcare”, the deep and long fissures in gendered medical discourses over the preceding seventy years give some indication of how we got where we are.

## Data availability

Data sharing is not applicable to this article, as no datasets were generated or analysed during the current study.

## References

[R1] Alexander S, Alexander S, Taylor B (2012). Primary maternal preoccupation: D.W. Winnicott and social democracy in mid-Twentieth-century Britain. History and Psyche: Culture, Psychoanalysis, and the Past.

[R2] Allen H, Miller P, Rose N (1986). Psychiatry and the construction of the feminine. The Power of Psychiatry.

[R3] American Medical Women’s Association (1953). MWIA Report of Council Meeting. Journal of the American Medical Women’s Association.

[R4] Beaumont C (2013). Housewives and Citizens: Domesticity and the Women’s Movement in England.

[R5] Beaumont C (2015). ‘What is a wife’? Reconstructing domesticity in post-war Britain before *The Feminine Mystique*. History of Women in the Americas.

[R6] Birmingham Feminist History Group (2005). Feminism as femininity in the Nineteen-Fifties?. Feminist Review.

[R7] Bliss K (1953). Forum on Family Relationships.

[R8] Bowlby J (1951). Maternal Care and Mental Health.

[R9] Bowlby J (1953). Child Care and the Growth of Love.

[R10] Bowlby J (1958). Can I Leave My Baby?.

[R11] Bradley H (1989). Men’s Work, Women’s Work.

[R12] Broverman IK, Broverman DM, Clarkson FE, Rosenktrantz PS, Vogel SR (1970). Sex-role stereotypes and clinical judgments of mental health. Journal of Consulting and Clinical Psychology.

[R13] Brown E (1948). Can Women Be Emancipated? Viola Klein Papers. MS1215/19/2.

[R14] Busfield J (1988). Mental illness as social product or social construct: A contradiction in feminists’ arguments?. Sociology of Health & Illness.

[R15] Buswell C, Glendinning C, Millar J (1992). Training girls to be low-paid women. Women and Poverty in Britain in the 1990s.

[R16] Campbell D (2016). Female doctors may be forced to quit over new contract, experts say. Guardian.

[R17] Caproni PJ (2004). Work/life balance: You can’t get there from here. The Journal of Applied Behavioral Science.

[R18] Carstairs GM (1963). This island now. British Medical Journal.

[R19] Cartwright A, Jefferys M (1958). Married women who work: Their own and their children’s health. British Journal of Preventive and Social Medicine.

[R20] Casson F (1959). It’s Healthy to Be Human.

[R21] Chesser E (1959). The Psychology of Everyday Living.

[R22] Clayton RW, Barton H (2011). Rhona rapoport: A critical biography of a pioneering work-family researcher. Journal of Applied Management and Entrepreneurship.

[R23] Cohen A (1960). Nursing a sick husband. Family Doctor.

[R24] Cooper C (2016). Hunt to ‘pause’ imposition of new junior doctors’ contracts. The i.

[R25] Dicks H (1955). The predicament of the family in the modern world. The Lancet.

[R26] Editorial (1951). Mental Health and the Mother. The Lancet.

[R27] Ehrenreich B, English D (1979). For Her Own Good: 150 years of the Experts’ Advice to Women.

[R28] Ericksson-Lihr Z (1953). Symposium from Austria, France, Italy and Finland. Journal of the American Medical Women’s Association.

[R29] European League for Mental Hygiene (1955). British Medical Journal.

[R30] Ford E (1953). Three children and a job: Blessing in disguise. Family Doctor.

[R31] Giles J (2004). The Parlour and the Suburb: Domestic Identities, Class, Femininity and Modernity.

[R32] Gill J, Jackson M (2007). Anne Sexton‘s poetics of the suburbs. Health and the Modern Home.

[R33] Haggett A (2016). Desperate Housewives, Neuroses and the Domestic Environment.

[R34] Haldane FP (1950). Some limitations of psychiatry. The Lancet.

[R35] Haldorssen I (1954). The menopause: its influence on working ability.

[R36] Halliwell M (2013). Therapeutic Revolutions: Medicine, Psychiatry, and American Culture, 1945–1970.

[R37] Halmos P (1953). Solitude and Privacy.

[R38] Hayward R, Jackson M (2007). Desperate housewives and model amoebae: The invention of suburban neurosis in inter-war Britain. Health and the Modern Home.

[R39] Hepler A (2000). Women in Labor: Mothers, Medicine, and Occupational Health in the United states, 1890–1980.

[R40] Herzberg D (2009). Happy Pills in America: From Miltown to Prozac.

[R41] Hilliard M (1954). The menopause and the working woman.

[R42] Hilliard M (1958). A Woman Doctor Looks at Love and Life.

[R43] Hirshbein L (2010). Sex and gender in psychiatry: A view from history. Journal of Medical Humanities.

[R44] Holdsworth A (1988). Out of the Doll’s House.

[R45] Holloway G (2005). Women and Work in Britain since 1840.

[R46] Houck J (2006). Hot and Bothered: Women, Medicine, & Menopause in Modern America.

[R47] Hubback J (1957). Wives Who Went to College.

[R48] Hutchin KC (1960). How to keep your husband alive. Family Doctor.

[R49] Iglehart AP (1980). Wives, work, and social change: What about the housewives?. Social Service Review.

[R50] Instone S (1948). The welfare of the housewife. The Lancet.

[R51] Jackson M (2013). The Age of Stress: Science and the Search for Stability.

[R52] Jackson M, Jackson M (2015). Men and women under stress: Neuropsychiatric models of resilience during and after the second world war. Stress in Post-War Britain, 1945–1985.

[R53] Jephcott P, Seear N, Smith J (1962). Married Women Working.

[R54] Jespersen I (1956). Untitled Paper.

[R55] Johnson L, Lloyd J (2004). Sentenced to Everyday Life: Feminism and the Housewife.

[R56] Kanter RM (1977). Work and Family in the United States: A Critical Review and Agenda for Research and Policy.

[R57] Klein V (1959). Working Wives: A Survey of Facts and Opinions Concerning the Gainful Employment of Married Women in Britain.

[R58] Klein V (1960). Working Wives.

[R59] Langhamer C (2005). The meanings of home in postwar Britain. Journal of Contemporary History.

[R60] Langhamer C (2016). Feelings, women and work in the long 1950s. Women’s History Review.

[R61] Lewis A (1953). Health as a social concept. The British Journal of Sociology.

[R62] Lewis J (1988). Women in Britain since 1945: Women, Family, Work and the State in the Post-War Years.

[R63] Lewis J, Smith HL (1990). Myrdal, Klein, women’s two roles and postwar feminism, 1945–1960. British Feminism in the Twentieth Century.

[R64] Long V (2011). The Rise and Fall of the Healthy Factory: The Politics of Industrial Health in Britain, 1914–60.

[R65] Luetkens C (1946). Women and a New Society.

[R66] Lunbeck E, Alexander S, Taylor B (2012). The narcissistic homosexual: Geneology of a myth. History and Psyche: Culture, Psychoanalysis, and the Past.

[R67] Lundberg F, Farnham M (1947). Modern Woman: The Lost Sex.

[R68] Lutz T (1996). “Sweat or die”: The hedonization of the work ethic in the 1920s. American Literary History.

[R69] Mace D (1948). Marriage Crisis.

[R70] McCarthy H (2016). Women, Marriage and Paid Work in Post-war Britain. Women’s History Review.

[R71] Mead M (1954). Some theoretical considerations on the problem of mother-child separation. American Journal of Orthopsychiatry.

[R72] Medical Women’s Federation (MWF) (1943). The menopause and after. The Lancet.

[R73] Medical Women’s Federation (MWF) (1958a). Interim report on the work of medical women qualified during the years 1933 and 1948.

[R74] Medical Women’s Federation (MWF) (1958b). Conference material.

[R75] Medical Women’s International Association (MWIA) (1954).

[R76] Medical Women’s International Association (MWIA) (1956a).

[R77] Medical Women’s International Association (MWIA) (1956b). Conference proceedings. British Medical Journal.

[R78] Meierhofer M (1956). Untitled Paper.

[R79] Mendes K (2010). Reading chatelaine: Dr. Marion Hilliard and 1950s women’s health advice. Canadian Journal of Communication.

[R80] Metzl J (2003). Prozac on the Couch: Prescribing Gender in the Era of Wonder Drugs.

[R81] Miller P, Miller P, Rose N (1986). Psychotherapy of work and unemployment. The Power of Psychiatry.

[R82] Mogey JM (1956). Family and Neighbourhood: Two Studies in Oxford.

[R83] Montreuil-Straus G (1953). The psychosomatic aspect of housework. Journal of the American Medical Women’s Association.

[R84] Morris JF (1961). When the manager comes home. Family Doctor.

[R85] Myrdal A (1941). Nation and Family: The Swedish Experiment in Democratic Family and Population Policy.

[R86] Myrdal A, Klein V (1956). Women’s Two Roles.

[R87] Oakley A (1984). Taking It Like a Woman.

[R88] Odlum D (1953). Bringing up baby the modern way. Family Doctor.

[R89] Odlum D (1953). Some special surveys in great Britain. Journal of the American Medical Women’s Association.

[R90] Odlum D (1956). Untitled Paper.

[R91] Penny L (2016). Maybe you should just be single. New Statesman.

[R92] Pollert A (1981). Girls, Wives, Factory Lives.

[R93] Reynaud E (1983). Holy Virility: The Social Construction of Masculinity.

[R94] Riesman D (1950). The Lonely Crowd.

[R95] Riley D (1979). War in the nursery. Feminist Review.

[R96] Riley D, Higonnet M, Jenson J, Michel S, Weisz M (1987). Some peculiarities of social policy concerning women in wartime and postwar Britain. Behind the Lines: Gender and the Two World Wars.

[R97] Rioch J (1955). Consideration of certain aspects of the dynamics of family life in the U.S.A.

[R98] Rowbotham S (1973). Women’s Consciousness, Man’s World.

[R99] Ruys AC (1947). Amsterdam Congress: Responsibilities in Reconstructing the World. Papers of the Medical Women’s Federation.

[R100] Ruys AC (1956). Resume.

[R101] Scrutton M (1956). The Push-me, Pull-you Woman. The New Statesman and Nation.

[R102] Serin S (1956). Untitled Paper.

[R103] Spence JC (1946). The Purpose of the Family.

[R104] Spencer S (2005). Gender, Work and Education in Britain in the 1950s.

[R105] Spring Rice M (1939). Working Class Wives: Their Health and Conditions.

[R106] Stanley J (1989). To Make Ends Meet: Women Over 60 Write About Their Working Lives.

[R107] Starkey P (2000). The feckless mother: Women, poverty and social workers in wartime and post-war England. Women's History Review.

[R108] Strange J-M (2012). In full possession of her powers: Researching and rethinking menopause in early twentieth-century England and Scotland. Social History of Medicine.

[R109] Taylor S (1938). The suburban neurosis. The Lancet.

[R110] Thomson M (2013). Lost Freedom: The Landscape of the Child and the British Post-War Settlement.

[R111] Tosoni-Dalai Y (1955). President’s address. Medical Women’s International Journal.

[R112] Van Andel-Ripke O (1954). Mother and housewife in the climacteric.

[R113] Van der Horst FCP (2011). John Bowlby—From Psychoanalysis to Ethology: Unravelling the Roots of Attachment Theory.

[R114] Vicedo M (2011). The social nature of the mother's tie to her child: John Bowlby's theory of attachment in post-war America. The British Journal for the History of Science.

[R115] Ward D (2010). They Cure in a Motherly Spirit: History of the Medical Women’s International Association.

[R116] Warren T (2015). Work—life balance/imbalance: The dominance of the middle class and the neglect of the working class. The British Journal of Sociology.

[R117] Welshman J (1996). In search of the “problem family”: Public health and social work in England and Wales, 1940–70. Social History of Medicine.

[R118] White AB (1941). Amber Bianco White, Worry in Women: Its Causes and Consequences.

[R119] Williams G (1945). Women and Work.

[R120] Wilson D (1949). Surveying the housewife. Public Health.

[R121] Wilson E (1980). Only Halfway to Paradise.

[R122] World Federation for Mental Health (1955).

[R123] Yudkin S, Holme A (1963). Working Mothers and Their Children: A Study for the Council for Children's Welfare.

[R124] Zweig F (1952). Women’s Life and Labour.

